# Comparisons of urine protein-to-creatinine ratios and their dynamic change patterns during labor at term between normal pregnant women and women with pregnancy induced hypertension

**DOI:** 10.7150/ijms.72926

**Published:** 2022-08-15

**Authors:** Pei-Yin Yang, Yi-Lun Tsai, Yu-Jun Chang, Po-Hui Wang

**Affiliations:** 1 Institute of Medicine, Chung Shan Medical University, No. 110, Section 1, Chien-Kuo North Road, Taichung, 40201, Taiwan Taichung, Taiwan; 2 Department of Obstetrics and Gynecology, Changhua Christian Hospital, No 135, Nanxiao Street, Changhua, 50094, Changhua, Taiwan; 3 Department of Delivery Room, Changhua Christian Hospital, No 135, Nanxiao Street, Changhua,50094, Changhua, Taiwan; 4 Department of Big Data Center, Changhua Christian Hospital, No 135, Nanxiao Street, Changhua,50094, Changhua, Taiwan; 5 School of Medicine, Chung Shan Medical University, Taichung, Taiwan; 6 Department of Obstetrics and Gynecology, Chung Shan Medical University Hospital, No. 110, Section 1, Chien-Kuo North Road, Taichung, 40201, Taiwan Taichung, Taiwan

**Keywords:** urine protein-to-creatinine ratio, pregnancy-induced hypertension, term delivery, dynamic change patterns

## Abstract

**Introduction:** To evaluate patterns of change in the urine protein-to-creatinine ratios (uPCRs) during labor at term between normal and women with pregnancy-induced hypertension (PIH).

**Methods:** This is an observational study in tertiary referral hospital, recruiting 269 women at term delivery in Taiwan from April 19, 2019 to April 18, 2021. uPCRs in four phases (latent, active, recovery and early postpartum) and related clinical data at delivery were collected. Multivariate analyses with a linear regression model were performed to analyze continuous variables after adjusting for clinical data between two groups.

**Results**: Based on exclusion criteria, 68 normal and 24 pregnant women with PIH were included. There were no differences in the uPCR or the proportion cases of uPCRs ≥ 300 mg/g between normal and PIH group in the four phases. There was a statistically significant tendency for the proportion of uPCRs ≥ 300 mg/g to increase from the latent to the early postpartum phase in both groups. The proportion of uPCRs ≥ 300 mg/g significantly increased from the active to the recovery phase and then declined from the recovery to the early postpartum phase in the normal group. Thus no differences in uPCRs cases change between any two phases in women with PIH, except the duration above stated.

**Conclusion:** This is the first study to demonstrate that uPCRs data are not different between normal pregnant and PIH groups during the course of labor, but it did show different dynamic change patterns throughout the labor phases.

## Introduction

Hypertensive disorders of pregnancy include forms of gestational hypertension, as pregnancy-induced hypertension (PIH), preeclampsia (PE; hypertension plus proteinuria), eclampsia, chronic hypertension with pregnancy and PE superimposed on chronic hypertension, as defined by the National High Blood Pressure Education Program Working Group on High Blood Pressure in Pregnancy 1. PE is a major complication during pregnancy and cause maternal and fetal death or other severe sequelae 2-4. The perinatal outcome of pregnant women with high blood pressure or preeclampsia with end organ damage is less favorable 5. In addition, proteinuria sometimes can be found in some normal pregnant women due to increased glomerular permeability, elevated plasma protein levels, decreased tubular protein absorption or renal hemodynamic alterations 6-10.

The urine protein-to-creatinine ratio (uPCR) is a reliable method for evaluating the amount of protein in urine samples from the general population and pregnant women 11-13. The 2013 American College of Obstetricians and Gynecologists (ACOG) Task Force on Hypertension in Pregnancy included a random urine uPCR ≥ 0.3 mg/mg (≥ 300 mg/g) as one of the diagnostic criteria for PE. Preeclampsia sometimes can be diagnosed in the absence of proteinuria if any of the following signs of end-organ dysfunction are present: elevated serum creatinine greater than 1.1 mg/dL or doubling of serum creatinine in the absence of other renal disease, thrombocytopenia (<100,000/μL), elevated liver transaminases greater than or equal to 2 times normal, pulmonary edema, or cerebral/visual symptoms 14 .The preeclampsia women mostly present with asymptomatic hypertension at a regular antenatal visit. The quickly confirmed with proteinuria is concerned. Some reviews have reported an optimal uPCR cutoff level between 0.30 and 0.35 mg/mg, with a sensitivity and specificity > 75% for proteinuria in PE 15,16. Another meta-analysis of spot uPCR measurement for diagnosing PE reported that a cutoff of 0.3 mg/mg (300 mg/g) had a sensitivity of 91% and a specificity of 90% 17.

A review reported that at 1 year after delivery, women with hypertensive disorders, including PIH and PE, have relatively more cardiovascular risks factors than women who had a normal pregnancy 18,19.A Canadian population-based study showed that women with a history of PE have the greatest increase in risk of end-stage renal disease followed by women with a history of PIH 20.

Pregnant women experience distinct changes in their cardiovascular and renal systems during labor. These changes have profound and lasting effects on women's health. Thus far, uPCR data have been used to define proteinuria for the diagnosis of PE without end organ damage, and not to routine monitor the renal function of pregnant women with PIH or PE. However, uPCRs are widely used for diagnosing and monitoring renal function in people with chronic kidney disease 21,22. Women with only PIH are defined to have no proteinuria throughout pregnancy and before labor. The conditions of uPCRs during labor have not been explored in women with PIH or in normal pregnant women. We hypothesized that uPCRs in the latent, active, recovery and early postpartum phases of labor at term are different between them. The purposes of this study were to compare the uPCRs in these phases, and the patterns of dynamic change in uPCRs from the latent to early postpartum phases between PIH and normal pregnant groups.

## Materials and method

Two hundred and sixty-nine pregnant Taiwanese women were recruited during labor at term at Changhua Christian Hospital from April 19, 2019 to April 18, 2021. Pregnant women were included in the study if they underwent vaginal delivery from 37 to 42 weeks of pregnancy; all were healthy with no complications, and some had PIH only found during routine antenatal visit. All were admitted while in the latent phase (cervical os dilation < 4 cm) and volunteered to participate in the study.

### Exclusion criteria

**Figure 1:**
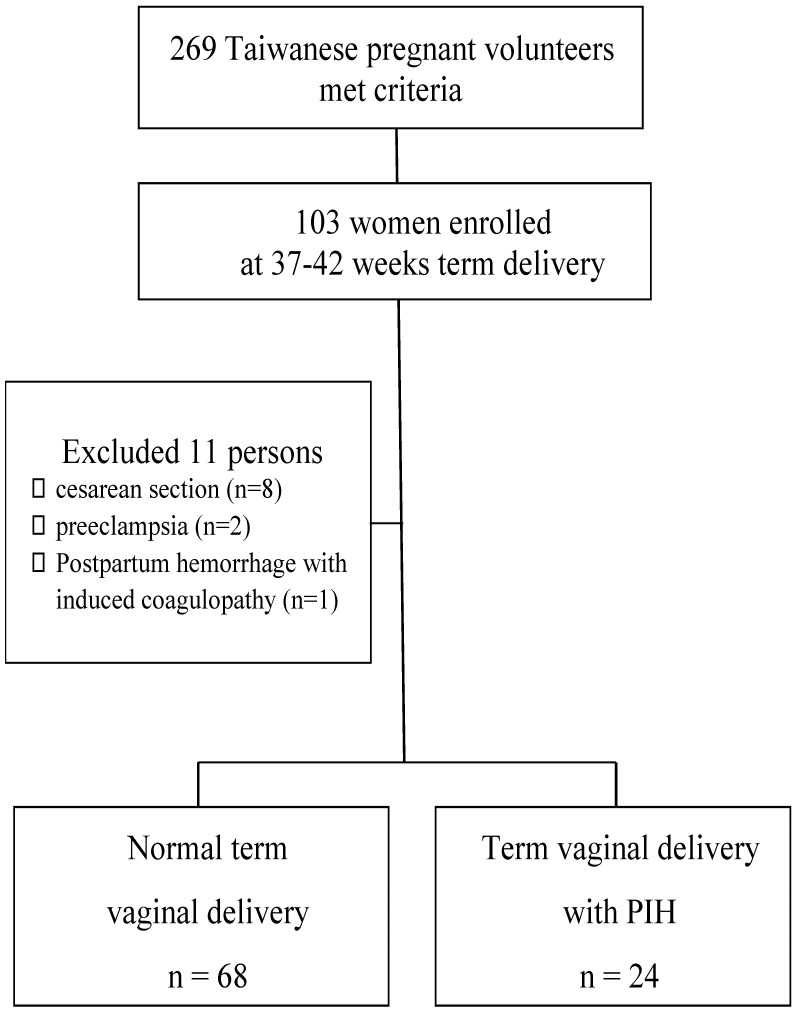
Flow chart of enrolled participants at 37-42 weeks vaginal delivery.

Pregnant women were excluded if they had preexisting hypertension (systolic blood pressure ≥ 140 mmHg and/or diastolic blood pressure ≥ 90 mmHg before 20 weeks of gestation); had a urinary tract infection diagnosed with clinical symptoms; had any underlying chronic medical conditions or pregnancy-associated complications, such as renal disease, diabetes mellitus, thyroid disease, cardiovascular disease or autoimmune disease; were taking medication that may affect renal function; admitted to the delivery room in active labor (cervical os dilation ≧4 cm) ; worse frequent blood pressure over 160/110 mmHg even under antihypertensive drug or lost most data (never received antenatal care in our hospital). Then, 103 women were enrolled at 37-42 weeks term delivery. Furthermore, eleven subjects were excluded because the urine data >300 mg/dL with postpartum headache matched PE (n = 2), cesarean section was performed (n = 8) or postpartum hemorrhage occurred due to disseminated intravascular coagulation and a trans-arterial embolism was done (n = 1; Figure 1). Finally, 24 women with PIH (antenatal routine visit conformed) were recruited as the case group, and 68 normal pregnant women were recruited as controls.

The uPCR is calculated as the amount of protein in the urine divided by the amount of creatinine in the same urine sample. The uPCR is a widely used metric to estimate daily protein excretion in the urine 23,24. Urine samples were collected via a midstream clean catch if neither ruptured amniotic membrane nor vaginal bleeding occurred. Otherwise, urine samples were collected by sterile catheterization. Urine samples were immediately sent to the hospital clinical laboratory for analysis using a DxC 700 AU Chemistry Analyzer (Beckman Coulter, Brea, CA, USA). uPCRs were measured at the following four stages during the course of labor for each participant: (1) the latent phase, from admission to < 4 cm cervical dilation; (2) the active labor phase, from≧4 cm dilation to full cervical dilation; (3) the recovery phase, within 4 hours of delivery; and (4) the early postpartum phase, from 4 to 58 hours postpartum. Three hundred and sixty-seven urine specimens were collected from 92 cases. Data were lost for one participant in the normal pregnancy group due to rapid progression through the course of labor from the latent phase to delivery. In addition, maternal and infant clinical characteristics were collected, including maternal age, gravidity, parity, gestational age, body mass index (BMI), and newborn birth weight and delivery method. BMI was classified as group 1 (< 18), group 2 (18-24) or group 3 (> 24) at admission. Delivery methods included normal spontaneous vaginal delivery and vacuum extraction delivery.

### Statistical analysis

We calculated the cases we need. When effect size (f2)= 0.15, power= 0.8, probability = 0.05 and predictor = 1 were set , the minimal required sample size is 64. (by G*Power version 3.1.9.7).

Student's *t* test, and the chi-square test or Fisher's exact test was used to compare the clinical characteristics between women with PIH and normal controls. The Mann-Whitney U method was used to compare uPCRs between two groups for non-parametric continuous variable analysis. uPCR data were also compared using the chi-square or Fisher's exact test for categorical analysis. The uPCR data were further compared between women with PIH and normal controls using multivariate analysis with a linear regression model to analyze continuous variables after adjusting for clinical data. Multivariate analysis using a logistic regression model was also performed to analyze categorical variables by defining an uPCR cutoff of 300 mg/g. These continuous and categorical variable analyses were regarded as the sensitivity analysis. Friedman test was used to compare changes in the uPCR in the latent, active, recovery and early postpartum phases in both groups for dependent, non-parametric, continuous variable analysis. Wilcoxon test was used for multiple pairwise comparisons, and Holm's test to adjust the *p* value to correct for multiple comparisons. Cochran's Q test, McNemar test and Holm's test were also performed for dependent categorical variable analysis. Moreover, the trends in uPCRs ≥ 300 were assessed in women with PIH and normal pregnant women using a logistic regression model. SPSS version 18.0 software (IBM, Armonk, NY, USA) and WinPepi Software, version 10.0 were used for statistical analyses, with the significance level set at 0.05 for the two-sided tests.

## Results

Clinical characteristics were compared between women with PIH and normal pregnant women, as shown in Table 1. The mean ages and their standard deviation (SD) of normal pregnant women and women with PIH were 31.3 ± 4.8 and 30.0 ± 4.0 years, respectively. The ranges of gravidity and parity were 1-8 and 0-3, respectively. The maternal gestational ages (± SD) of normal pregnant women and women with PIH were 39.4 ± 0.9 and 39.0 ± 1.0 weeks, respectively. There were no significant differences in the distribution of any of the clinical characteristics between women with PIH and normal pregnant women.

The medians (ranges) of uPCR in the latent, active, recovery and early postpartum phases of all 92 pregnant women were 183.7 (84.2-675.5), 207.1 (78.5-4786.0), 345.0 (124.8-3994.4) and 259.6 (62.6-2212.4) mg/g, respectively. The medians (ranges) of uPCR in the latent phase were 182.1 mg/g (101.5-675.5) in normal pregnant women and 194.8 mg/g (84.2-567.0) in women with PIH. There were no differences in uPCRs in the latent phase between normal pregnant women and women with PIH (*p* = 0.575, Table 1). uPCRs were also not significantly different in the active, recovery and early postpartum phases between normal pregnant women and women with PIH (*p* = 0.458, 199.7 vs 220.0 mg/g; *p* = 0.499, 345.0 vs 365.7 mg/g and *p* = 0.384, 248.1 vs 321.1 mg/g, respectively; Table 1). If uPCR 300 mg/g was determined as a cutoff level, 11.8% (8/68) of normal pregnant women had a uPCR ≥ 300 mg/g in latent phase, whereas in the active, recovery and early postpartum phases, 19.1% (13/68), 60.3% (41/68) and 36.8% (25/68) of normal pregnant women had a uPCR ≥ 300 mg/g, respectively (Table 2). In women with PIH, 16.7% (4/24) had a uPCR ≥ 300 mg/g in the latent phase, and 33.3% (8/24), 66.7% (16/24) and 54.2% (13/24) had a uPCR ≥ 300 mg/g in the active, recovery and early postpartum phases, respectively (Table 2).

In addition to a univariate analysis of the effect of PIH on uPCRs in the latent, active, recovery and early postpartum phases, clinical characteristics were also included to assess the associations between these variables and uPCRs during the course of labor via multivariate non-parametric continuous variable analysis with a linear regression model. PIH was not associated with uPCR in any phase of labor. Although PIH was not associated with uPCR in the latent phase (*p* = 0.907; B estimate = 3.06), a gravidity of 2, but not a gravidity ≥ 3, was found to be significantly associated with uPCR in the latent phase, compared with a gravidity of 1 (*p* = 0.004; B estimate = 111.33; Table 3).

Moreover, BMI group 3 was also associated with uPCR in the latent phase, compared with BMI group 2 (*p* = 0.038; B estimate = -67.26). In a multivariate categorical variable analysis with a logistic regression model, PIH was not associated with uPCR at any phase of labor.

Furthermore, a gravidity of 2, but not a gravidity ≥ 3, was found to be significantly associated with uPCR in the latent phase, compared with a gravidity of 1 (*p* = 0.029; odds ratio [OR] = 9.80, 95% confidence interval (95% CI=1.26-76.09; Table 4). In the early postpartum phase, a gravidity of 2 was found to be significantly associated with uPCR, compared with a gravidity of 1 (*p* = 0.021; OR = 8.37, 95% CI = 1.38-50.76). Only a parity of 1 was significantly associated with a reduced proportion of patients with a uPCR ≥ 300 mg/g, compared with a parity of 0 (*p* = 0.046; OR = 0.18, 95% CI = 0.03-0.97). Newborn body weight tended to be associated with uPCR (*p* = 0.042; OR = 1.00, 95% CI = 0.99-1.00, Table 4) in the early postpartum phase. The logistic R^2^ of model in the latent, active, recovery, postpartum phase excluded discrete value >1000 is 0.256, 0.252, 0.308 and 0.192; linear R^2^ of the model is 0.218, 0.143, 0.341, 0.123.

Although conflicting results were obtained regarding gravidity, parity, BMI and newborn body weight, the sensitivity analysis showed that PIH was not associated with uPCR in any phase of labor in either univariate or multivariate analysis of continuous or categorical variable.

The dynamic change patterns of uPCR during the course of labor were further evaluated. The uPCRs were elevated, and reached a peak during the recovery phase after term delivery compared with uPCRs in the latent phase in both normal pregnant and PIH groups.

**Figure 2:**
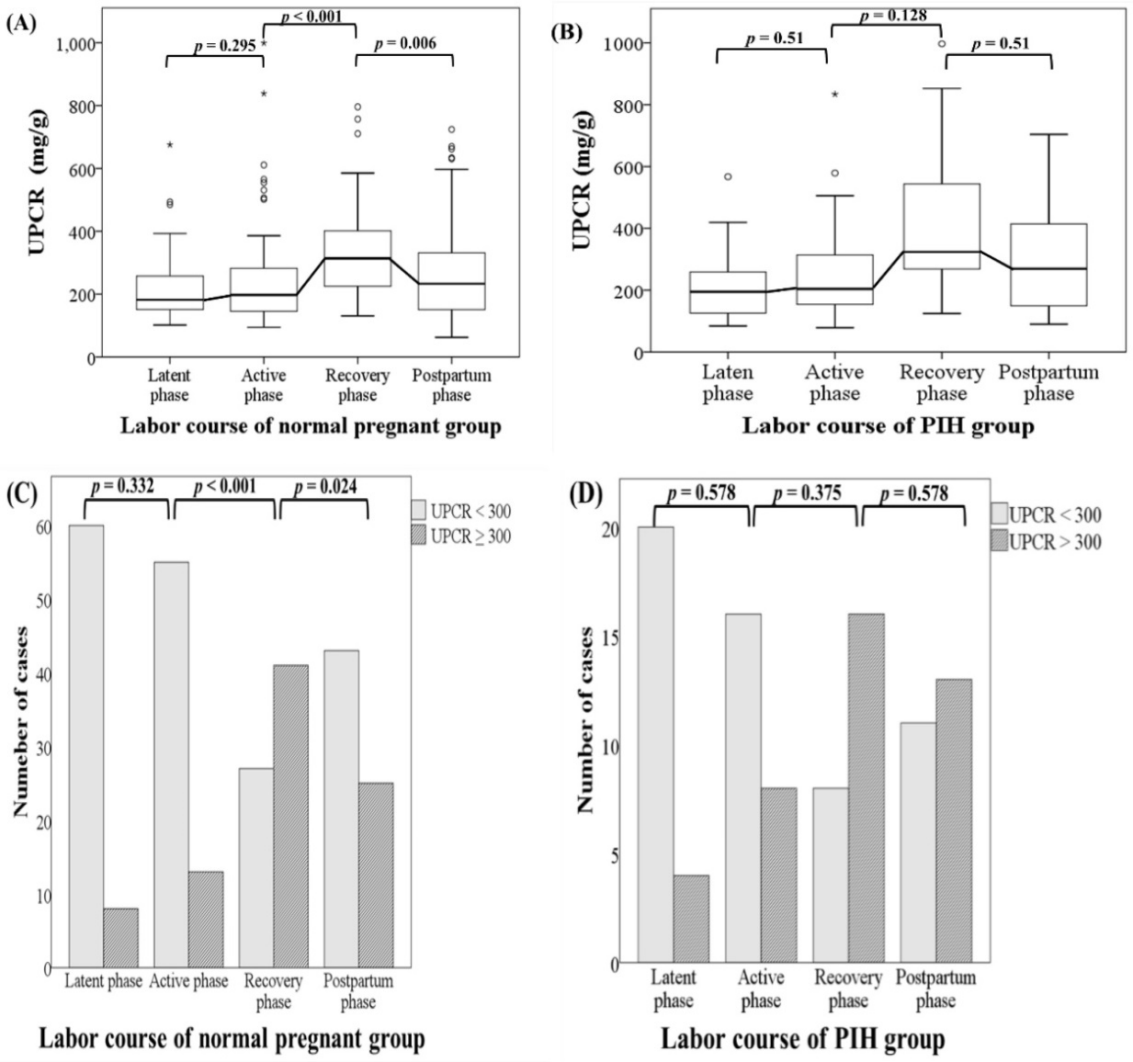
The dynamic change patterns in uPCR and the proportions of uPCRs ≥ 300 mg/g in the latent, active, recovery and early postpartum phases during the course of labor at term delivery in normal pregnant women and women with pregnancy-induced hypertension. (A) Normal pregnant women. There was no significant difference in uPCR from the latent phase to the active phase. Thereafter, uPCR increased significantly from the active phase to the recovery phase and decreased significantly from the recovery phase to the early postpartum phase. (B) Women with pregnancy-induced hypertension. No obviously significant differences in uPCR were found from the latent phase to the early postpartum phase. Statistical analyses of dependent continuous variables were performed using Friedman, Wilcoxon and Holm's tests. uPCR, urine protein-to-creatinine ratio.(C) Normal pregnant women. There was no significant difference in the proportions of uPCRs ≥ 300 mg/g from the latent phase to active phase. Thereafter, the uPCR increased significantly from the active phase to the recovery phase and then decreased significantly from the recovery phase to the early postpartum phase. (D) Women with pregnancy-induced hypertension. No obviously significant differences were found in the proportions of uPCRs ≥ 300 mg/g from the latent phase to the early postpartum phase. Statistical analyses of dependent categorical variables were performed using Cochran's Q, McNemar and Holm's tests. uPCR, urine protein-to-creatinine ratio; PIH, pregnancy-induced hypertension.

The trends in the proportion of patients with a uPCR ≥ 300 were statistically significant in both the normal and PIH groups (*p* < 0.001 and *p* = 0.002, respectively). This implied that the number of patients with an uPCR ≥ 300 tend to increase sequentially from the latent phase to the early postpartum phase in both normal pregnant women and women with PIH. Although there was no significant difference from the latent phase to the active phase, in normal pregnant women, uPCRs (including real uPCRs data and the proportions of uPCR ≥ 300) increased significantly from the active phase to the recovery phase and then decreased significantly from the recovery phase to the early postpartum phase in both dependent continuous and categorical variable analyses. However, there were no obviously significant changes in uPCRs from the latent phase to the active phase, from the active phase to the recovery phase, and from the recovery phase to the early postpartum phase in women with PIH in analysis of either variable type. The dynamic change patterns of uPCRs during the latent, active, recovery and early postpartum phases at term delivery were different between normal pregnant women and women with PIH and are shown in Figure 2.

## Discussion

The uPCRs data in the latent, active, recovery and early postpartum phases were not different between normal pregnant women and women with PIH at term delivery. The uPCR over 300 mg/g is common during term labor, not related to hypertension. However, the dynamic change pattern of uPCRs in women with PIH does differ with that in normal pregnant women.

### Strength and limitation

There are several unique characteristics of this study, including the prospective study design, the repeated measurement of uPCRs during the course of labor at term and enrolled women with PIH. Our study may be the first to simultaneously assess uPCRs in the four phases of term delivery in normal and PIH pregnant women and to compare uPCRs between these two groups. Another novel finding is that the dynamic uPCR change patterns during the course of labor at term are different between normal pregnant women and women with PIH.

There were four main limitations of this study. First, some urine specimens were obtained by self-voiding, especially in the latent and early postpartum phases, because the participants refused catheterization. This may have caused contamination of the urine samples and led to inaccurate results. Second, the PIH sample size was relatively small. This occurred because several participants in the PIH group shifted to cesarean sections due to prolonged labor (termination due to frequently worse blood pressure over 160/110 mmHg and unfavorable Bishop score). Third, because women with severe PIH required cesarean section may be excluded. In addition, only patients with cervical dilation less than 4 cm were included, and selection bias may present. Fourth, conflicting results were found for the BMI group and newborn body weight in linear regression and logistic regression models. It was also noted in the data of R^2^ in our model. However, the* p* values (*p* = 0.038 and 0.042, respectively) were near the significance limit of two-sided tests of significance. In addition to hypertension, more cases may be necessary to further investigate associations between uPCRs and BMI, newborn body weight, gravidity and parity in normal pregnant women and women with PIH in future studies.

### Interpretation

In our study, a uPCR ≥ 300 mg/g occurred at a frequency of 11.8% (8/68) in the latent phase, 19.1% (13/68) in the active phase, 60.3% (41/68) in the recovery phase and 36.8% (25/68) in the early postpartum phase in normal term delivery women. Tanamai et al. reported that 39.1% of women not in labor at term pregnancy had a uPCR ≥ 300 mg/g 23. A uPCR ≥ 300 mg/g is common in term normal pregnancy. Increased basement membrane permeability at the glomeruli, increased glomerular flow due to cardiac output elevation, increased emotional stress associated with elevated levels of circulating catacholamines and a decrease in renal tubular protein reabsorption are possible causes of proteinuria during labor 5,8,24,25.

Based on the results of univariate and multivariate analyses of continuous and categorical variables, neither the uPCRs nor the proportions of patients with a uPCR ≥ 300 mg/g were different in the four phases between our two study groups. This was regarded as a sensitivity analysis, thus confirming our findings that uPCRs do not differ during the course of labor between the two groups. It implies that the duration from presenting with elevted blood pressure to early postpartum is not long enough to cause significant proteinuria in PIH women as compared to normal pregnant women. Therefore, proteinuria may not be associated with or caused by hypertension during pregnancy, because no differences in uPCRs were observed between normal pregnant women and women with PIH in this study.

Through continuous variable analysis using a linear regression model, we found that uPCRs were significantly higher in patients with a gravidity of 2 compared with those with a gravidity of 1, and in BMI group 3 compared with BMI group 2 only in latent phase. However, uPCRs were not associated with hypertensive episodes, maternal age, parity, gestational age, newborn body weight or delivery method in any phase during the course of abor. In the logistic regression model for categorical variable analysis, the OR of a uPCR ≥ 300 mg/g increased in patients with a gravidity of 2 compared with those with a gravidity of 1 in the latent and early postpartum phases. The number of women in our study with a parity of 0 was 41, and the number of women with a gravidity of 1 was only 31, suggesting that more women may have had a gravidity of 2 or more but a parity of 0. If women with a gravidity of 2 or more terminated their pregnancies early, the influence of gravidity on pregnancy-induced changes may be lessened. In contrast, women with a parity of 1 exhibited a lower risk of an uPCR ≥ 300 mg in the early postpartum phase compared with those with a parity of 0 in the logistic regression model. Because the sample size was relatively small, the significance of gravidity and parity requires further investigation. The perinatal outcomes in ours study only included newborn body weight and Apgar score; the uPCR data seems to be not related to the newborn weight in our study (Table 3 and 4). However, larger sample size is necessary to delineate the relationship between proteinuria and perinatal outcome in future study.

In normal pregnant women, the uPCR reached its highest value in the recovery phase, after which it decreased significantly in the early postpartum phase using dependent continuous (real uPCR data) and categorical (the proportions of uPCR ≥ 300) variable analyses in this study. However, in women with PIH group, there were no obvious differences in uPCRs between the latent and active phases, between the active and recovery phases as well as between the recovery and early postpartum phases. Garber et al. proposed the now widely accepted term, glomerular capillary endotheliosis, as a pathological finding of PE; however, this later found to be present also in women with gestational hypertension and, similarly, in normal pregnancies women. PE may be an extreme adaptational process, rather than a separate abnormal condition 26. In our study, the dynamic change patterns of uPCRs in the PIH group may reflect this phenomenon, i.e. the uPCR reached its highest value in the recovery phase and did not decrease significantly in the early postpartum phase. Some studies had confirmed renal glomerular filtration flow decreased in these hypertension, preeclampsia cases due to endothelial damage 27. However, as pevious statement, increased basement membrane permeability at the glomeruli, increased glomerular flow due to cardiac output elevation, stress related with elevated levels of catacholamines and a decrease in renal tubular protein reabsorption are possible causes of proteinuria during labor. We inferred the difference of uPCR data and proportion >300 dynamic change in PIH and normal group is influenced by these two mechanism and these two seem to cancel each other. In normal cases, the renal glomerular endothelial condition is well function, it can well reflect basement membrane permeability, increased glomerular flow, and decreased renal tubular protein reabsorption during labor. Therefore, the uPCR (meaning the condition of proteinuria) changed obviously and significantly returrned to more normal status in the processing of labor course. Nevertheless, in PIH cases, the renal glomerular endothelial condition is damaged with decreased glomerular filtration flow so it cannot appear dramatic change of uPCR data during whole labor course observed in our study. This implied that women with PIH may have glomerular changes usually not significantly returned in postpartum but subtle endothelial injury may persit probably several years after delivery; the uPCR however returned to normal very quickly in normal group. Although uPCRs were not obviously different between the normal pregnant and PIH groups, the different dynamic change patterns exist, and these may arouse our alertness for this pathogenesis to help prevent the occurrence of complications. It is worth investigating whether persistently high or different change pattern uPCRs in women with PIH affect long-term renal function in the future. Therefore, participants with PE or severe gestaional hypertension (often over 160/100 mmHg) should be enrolled in future studies.

This is the first study to demonstrate that there are no differences in uPCRs and the proportions of women with uPCR ≥ 300 mg/g in the latent, active, recovery and early postpartum phases by univariate and multivariate methods in continuous and categorical variable analyses with sensitivity analysis significance between normal pregnant women and women with PIH. Although the trends in the number of women with an uPCR ≥ 300 mg/g were statistically significant in both the normal pregnant and PIH groups, we report the novel finding that different dynamic change patterns of uPCRs occurred between the two groups.

## Figures and Tables

**Table 1:** Characteristics of women with pregnancy-induced hypertension and normal pregnant women

Characteristics	Normal pregnant women (n = 68)	Women with PIH (n = 24)	*p* value
Age (years, mean ± SD)	31.3 ± 4.8	30.0 ± 4.0	0.233
Gravidity			0.436
1	31	17	
2	20	4	
3	9	2	
4	6	1	
5	1	0	
8	1	0	
Parity			0.201
0	41	20	
1	23	3	
		
2	3	1	
		
3	1	0	
Gestational age (weeks, mean ± SD)	39.4 ± 0.9	39.0 ± 1.0	0.104
Body mass index			0.104
2	13	1	
3	55	23	

Statistical analysis: Student's *t* test and chi-square or Fisher's exact test. Participants were classified by body mass index as group 1 (< 18), group 2 (18-24) and group 3 (> 24). PIH, pregnancy-induced hypertension, SD, standard deviation.

**Table 2:** Comparisons of urine protein-to-creatinine ratios during labor at term between normal pregnant women and women with pregnancy-induced hypertension

uPCR during the course of labor	Normal pregnant women (n = 68)	Women with PIH (n = 24)	*p* value	OR^*^ (95% CI)
**Latent phase**				
median (range)	182.1 (101.5-675.5)	194.8 (84.2-567.0)	0.575	
uPCR < 300 mg/g	60	20	0.504	1.00
uPCR ≥ 300 mg/g	8	4		1.50 (0.41-5.52)
**Active phase**				
median (range)	199.7 (94.4-1797.4)	220.0 (78.5-4786.0)	0.458	
uPCR < 300 mg/g	55	16	0.154	1.00
uPCR ≥ 300 mg/g	13	8		2.12 (0.75-6.00)
**Recovery phase**				
median (range)	345.0 (130.6-3994.4)	365.7 (124.8-2067.7)	0.499	
uPCR < 300 mg/g	27	8	0.580	1.00
uPCR ≥ 300 mg/g	41	16		1.32 (0.50-3.50)
**Early postpartum phase**				
median (range)	248.1 (62.6-2212.4)	321.1 (90.0-2000.0)	0.384	
uPCR < 300 mg/g	43	11	0.137	1.00
uPCR ≥ 300 mg/g	25	13		2.033 (0.792-5.215)

Statistical analysis: Mann-Whitney U test and chi-square or Fisher's exact test ^*^OR, odds ratio for the odds of uPCR≥ 300 mg/g to uPCR < 300 mg/g, normal pregnant women as a reference group. uPCR, urine protein-to-creatinine ratio; PIH, pregnancy-induced hypertension; 95% CI, 95% confidence interval.

**Table 3:** Multivariate analysis of the relationships between urine protein-to-creatinine ratio and hypertension and other important variables during labor at term delivery using a linear regression model

uPCR during the course of labor	Latent phase uPCR B estimate *p*	Active phase uPCR B estimate *p*	Recovery phase uPCR B estimate *p*	Postpartum phase uPCR B estimate *p*
PIH vs normal	3.06 0.907	189.49 0.181	-11.70 0.940	131.62 0.257
Age	-0.30 0.905	-7.14 0.597	6.04 0.686	0.49 0.965
Gravidity				
gravidity 2 vs gravidity 1	111.33 0.004^*^	-70.72 0.725	152.49 0.495	127.77 0.440
gravidity ≥ 3 vs gravidity 1	48.13 0.219	-22.16 0.914	132.76 0.559	38.52 0.819
Parity				
parity 1 vs parity 0	-72.47 0.055	-48.72 0.808	-245.71 0.270	-212.54 0.198
parity ≥ 2 vs parity 0	-46.49 0.436	-75.28 0.813	37.12 0.916	-296.39 0.259
Gestational age	-5.88 0.658	36.774 0.604	-3.94 0.960	62.38 0.285
BMI group^#^				
3 (> 24) vs 2 (18-24)	-67.26 0.038^*^	-9.82 0.954	-333.63 0.082	46.81 0.740
Newborn weight	-0.01 0.782	0.19 0.289	-0.06 0.754	-0.10 0.487
Delivery method				
VED vs NSD	22.43 0.372	88.92 0.507	176.64 0.236	-26.44 0.810

Statistical analysis: linear regression model, uPCR was regarded as continuous variable ^*^uPCR in latent phase: gravidity of 2 vs gravidity of 1, B estimate: 111.33, 95% CI: 36.75-185.91, *p* = 0.004; BMI 3 (> 24) vs 2 (18-24), B estimate: -67.26, 95% CI: -130.8- -3.67, *p* = 0.038 ^#^Participants were classified by body mass index (BMI) as group 1 (< 18), group 2 (18-24) and group 3 (> 24). uPCR, urine protein-to-creatinine ratio; PIH, pregnancy-induced hypertension; VED, vacuum extraction vaginal delivery; NSD, normal spontaneous vaginal delivery.

**Table 4:** Multivariate analysis of the relationships between urine protein-to-creatinine ratio and hypertension and other important variables during labor at term delivery using a logistic regression model

uPCR in the course of labor	Latent phase uPCR		Active phase uPCR		Recovery phase uPCR		Postpartum phase uPCR	
	OR	*p*	OR	*p*	OR	*p*	OR	*p*
PIH vs normal	2.45	0.278	1.91	0.311	0.90	0.856	2.81	0.078
Age	0.95	0.585	0.99	0.836	0.93	0.237	0.99	0.861
Gravidity								
gravidity 2 vs gravidity 1	9.80	0.029^*^	2.31	0.323	1.16	0.862	8.37	0.021^*^
gravidity ≥ 3 vs gravidity 1	1.97	0.620	1.25	0.819	1.21	0.815	3.56	0.137
Parity								
parity 1 vs parity 0	0.39	0.370	0.19	0.086	0.31	0.148	0.18	0.046^*^
parity ≥ 2 vs parity 0	u.a.	u.a.	u.a	u.a.	u.a.	u.a.	0.31	0.400
Gestational age	1.23	0.615	0.92	0.7914	0.78	0.395	1.84	0.050
BMI group^#^ 3 vs 2	0.19	0.058	0.42	0.260	0.47	0.302	0.66	0.534
Newborn weight	1.00	0.327	1.00	0.219	1.00	0.791	1.00	0.042^*^
Delivery method VED vs NSD	1.07	0.931	1.14	0.825	2.02	0.196	1.43	0.500

Statistical analysis: logistic regression model, uPCR regarded as a categorical variable by defining uPCR ≥ 300 mg/g as abnormal ^*^uPCR assessed by the proportion of uPCRs ≥ 300, and the OR and 95% CI of uPCR ≥ 300 for gravidity 2 vs gravidity 1 (OR: 9.80, 95% CI: 1.26-76.09, *p* = 0.029) in latent phase. uPCR in early postpartum phase gravidity 2 vs gravidity 1 (OR: 8.37, 95% CI: 1.38-50.76, *p* = 0.021); parity 1 vs parity 0 (OR: 0.18; 95% CI: 0.03-0.97, *p* = 0.046); newborn body weight (OR: 1.00, 95% CI: 0.99-1.00, *p* = 0.042) ^#^Participants were classified by body mass index (BMI) as group 1 (< 18), group 2 (18-24) and group 3 (> 24). uPCR, urine protein-to-creatinine ratio; OR, odds ratio; 95% CI, 95% confidence interval; PIH, pregnancy-induced hypertension; VED, vacuum extraction vaginal delivery; NSD, normal spontaneous vaginal delivery; u.a., unavailable.

## Abbreviations

PIH: pregnancy induced hypertension
PCRs: urine protein-to-creatinine ratios
PE: preeclampsia
